# Hospital Meetings, &c.

**Published:** 1899-05-13

**Authors:** 


### HOSPITAL MEETINGS, &C.

### HOSPITAL FOR SICK CHILDREN, GREAT ORMOND STREET.

The festival dinner in aid of the Hospital for Sick Children, Great Ormond Street, was held on Wednesday evening, 3rd inst., at the Hotel Cecil. Viscount Peel was in the chair, and amongst those supporting him were the Duke of Fife (president of the hospital), Viscount Duncannon, C.B., Sir John Wolfe Barry, Lord Aberdare, Sir Cameron Gull, Mr. Sydney Gedge, M.P., Sir W. Kynsey, Mr. Harold Reckitt, M.P., Mr. T. Warrington, Q.C., Sir Trevor Lawrence, Lord Crofton, Lord Tewkesbury, and Mr. Adrian Hope (secretary to the hospital).

The toast of "The Queen" having been duly honoured, Sir John Wolfe Barry proposed "The Health of the Prince and Princess of Wales and other Members of the Royal Family." There was no matter, he said, which touches the welfare of every class of this country which is not dear to the heart of the Prince and Princess of Wales—(hear, hear)—and every member of the Royal Family. The toast of the Prince demanded very special attention. We must all recognise the beneficent urgency with which he had pleaded the cause of the hospitals of London. (Applause.) In the case of this particular hospital the Princess had always shown by her patronage and gracious help her womanly love for little ones. (Applause.)

Lord Peel, in proposing the toast of the evening—"Prosperity to the Hospital for Sick Children"—said that he was very well aware of the responsibility which attached to any man who occupied that chair. He had in his mind some of those who had preceded him in that capacity. But it did not need the pathos and the feeling of a Charles Dickens, nor the eloquence of bishops, nor the great attraction that had been brought to bear upon that subject by members of the Royal family to advocate that cause, because it was one that pleaded for itself. (Applause.) It was a very singular thing to reflect that fifty years ago there was no such thing in the kingdom as a children's hospital. Fifty years ago—or thereabouts—it entered into the mind of some public-spirited individuals to institute a hospital of that character, and their hospital was the first of its kind. (Hear, hear.) In 1852 there had been only 20 beds, now there were something like 250. Their example had been followed, and there were now in London 16 hospitals for children. This was a great advance, but the very advance had brought about a great responsibility and a great danger. He hardly liked to use the word competition in connection with a hospital, but it was a fact that there were now immense demands made upon the charity of the people of the metropolis. He hoped that the streams of subscriptions that were flowing into a well-known fund would never dry up the little subsidiary rills that flowed into the treasury of the Hospital for Sick Children. He refused to believe that we should ever have recourse to State control of our hospitals or the municipalisation of our charities. Every year their hospital needed £5,000 in voluntary contributions in order to avoid the closing of cots. He was sure that if this fact were generally known that would be enough to prevent such a lamentable occurrence. An additional sum of £20,000 was required in order to carry out the work of enlarging the hospital, and thus giving better accommodation for the nurses. Amid great applause Lord Peel read a letter from Sir Theodore Martin enclosing a cheque for £1,000. This was to endow a "Helen Faucit" cot in memory of his wife, whose loss he had so lately to deplore.

Lord Duncannon, who gave the toast of "The Committee of Management," said that all who subscribed to the funds of that hospital might rest assured that their money was doing a great deal of good. (Applause.)

Mr. John Murray, vice-chairman of the Committee of

Management, responded to the toast with an eloquent speech. He reminded his hearers that the children of the poor were as dear to them as "ours are to us." (Applause.) He warmly eulogised Mr. Arthur Lucas for the self-sacrificing way in which he had worked as chairman of the committee, and regretted that he was not present.

The Duke of Fife proposed "The Health of the Chairman." The remaining toast, that of "The Medical Officers," was proposed by Lord Aberdare, and responded to by Mr. John H. Morgan, senior surgeon.

During the evening the Secretary (Mr. Adrian Hope) announced that £4,700 had been subscribed. This included 25 guineas from the chairman, and £100 from the Duke of Fife.

### KING'S COLLEGE HOSPITAL.

The annual festival dinner of the King's College Hospital was held on Wednesday (3rd inst.) at the Hotel Cecil, the Right Hon. and Right Rev. The Lord Bishop of London presiding. Among the guests present were Lieut.-General Moncrieff, Mr. Charles Awdry (treasurer), Mr. A. H. A. Morton, M.P., Mr. J. Lowles, M.P., Lieut.-Colonel and Sheriff Probyn, the Rev. Dr. Wace, the Rev. Dr. Robertson (principal of King's College), the Rev. Dr. Robins, Mr. William Rhose, M.B., F.R.C.S., Mr. H. A. Giffard, Q.C., Dr. Burney Yeo, Mr. F. O. Bevan, Dr. Ferrier, F.R.S., Professor Douglas, Dr. Hayes, Sir Hugh Beevor, Bart., M.B., Mr. Charles Plumtre Johnson, Dr. Urban Pritchard, Mr. C. E. Layton, Mr. Samuel Twining, Mr. H. H. Twining, Mr. Harvey Twining, Mr. Loftus Long, Professor Hughes, M.B., F.R.C.S., the Rev. Canon Porter, &c.

The toasts of "The Queen" and the "Prince and Princess of Wales, and the other Members of the Royal Family," having been honoured,

The Chairman, in proposing "Success to King's College Hospital," said the institution was one that not only rendered great service to the community at large as a hospital, but as a medical school did a great educational work in which they were all deeply interested. When he was in a gloomy state of mind, possibly due to the fact that someone had said something particularly offensive about him in the newspaper—(laughter)—he found comfort in the hospital, not, perhaps, a cheerful subject of contemplation in itself, but cheering as showing the general tendencies of the age, and tending to change pessimism into optimism. (Applause.) There was nothing that restored confidence in our race and nature, and proved that the world was not so bad as they were sometimes inclined to think it, and that civilisation was not an imposture and a sham. One of the tests, however rude and rough, which showed how far the human race had travelled, was the growing respect for human life, nowhere so evident as in those great hospitals which did so much to produce a sense of solidarity between class and class, by showing that the community was anxious to provide for the poor, not merely as much as could be obtained by the wealthy, but more. (Applause.) The hospitals also had a great educational effect on the nation. (Hear, hear.) They were now spread all over the country, but nowhere were they so large, and nowhere were they so much used, as in this great metropolis, and nowhere, he ventured to say, were they so well managed. (Applause.) King's College Hospital had now existed for sixty years, doing most magnificent work, and during that time it had benefited no fewer than a million and a half of patients—men, women, and children. (Applause.) He rejoiced to think that it was the tendency of our English race that such institutions should not be pitifully doled out by the hand of the State, not tied up with tape, nor smothered with red ink—(laughter)—but that these great institutions should be managed by volunteers and that they should owe their resources to voluntary efforts and should be commanded by a medical staff who gave their services gratuitously. (Applause.) He was sure they were all equally anxious for the welfare of these institutions, which were the pride of our country. (Renewed applause.) King s College Hospital was not so badly off as some institutions, but it required an additional £1,000 or £1,500 a-year more income. It was their object to obtain that sum, and he hoped that they would accomplish their desire. At the present time there was an immediate demand for £3,000 for the reflooring of the wards, and he was quite sure that the required amount would be forthcoming as they had every confidence in the management of this institution and were well aware of the good work it was doing. (Applause.)

The toast was enthusiastically received.

Dr. Robertson (principal of King's College) responded. He said that the number of beds occupied during the period under review exceeded the average of former years. The results of the treatment of in-patients, eliminating those now in the hospital and those brought in dead or nearly dying, showed that four-fifths of this class of patients had left the hospital entirely cured or very substantially relieved. (Applause.) As regarded the out-patients, they came for treatment from the extreme southern, eastern, and western limits of London, which was a high tribute to King's College Hospital. (Applause.) The balance sheet showed a deficiency in the revenue account of £965, but so far as the capital account was concerned, they were £3,800 better off than they were at the beginning of the year. (Applause.) To meet the expenditure £18,000 was required per annum. The invested funds brought in about one-twelfth of that amount; legacies, which were naturally a very precarious source of income, one-third; the Prince of Wales's Fund, the Hospital Sunday Fund, and the Hospital Saturday Fund one-sixth; annual subscriptions, one-seventh; and donations of friends, who assembled on occasions like the present, about one-fifth. The annual subscriptions had not diminished to any extent, as had in some quarters been anticipated, by the Prince of Wales's Fund. (Hear, hear.)

Lieut.-Colonel and Sheriff Probyn submitted "The National Services—The Church, the Law, and the Naval and Military Forces."

The Rev. Dr. Wace, Mr. H. A. Giffard, Q.C., and Lieutenant General Moncrieff having replied,

Mr. C. P. Johnson proposed the toast of "The Medical Staff of the Hospital."

Dr. Burney Yeo, in acknowledgment, pointed out that King's College Hospital inaugurated the system of nursing in the army, and it was also the first of the metropolitan hospitals to give a home to antiseptic surgery. (Applause.)

Mr. Charles Awdry (treasurer), in submitting "The Chairman," announced that subscriptions had been received to the amonnt of £2,190 18s. 6d. (Applause.) He mentioned that £300 had been received from Messrs. W. H. Smith and Sons, 100 guineas from Mr. C. P. Johnson, 100 guineas from "C. W.," £100 from the Hon. W. F. D. Smith, M.P., and £100 from Mr. C. Pearce Serocold. (Applause.)

The Chairman having responded, the proceedings terminated.

### BRITISH HOME FOR INCURABLES.

The annual meeting of the British Home for Incurables was held at the Cannon Street Hotel on Tuesday, 9th inst., Lord Amherst presiding.

The Secretary, Mr. R. G. Salmond, read the report presented by the Board of Management, which congratulated the subscribers on another year's successful work. During the year the annual subscriptions increased, reaching a total of £3,156. Donations fell off somewhat as compared with the previous year, when three separate donations of £1,000 each were received; but not counting these, the donations came up to the usual average; there being, in addition, £1,355 towards the completion fund. The total income for the year was £15,314, including £5,505 from legacies—an increase in the latter item of £700 over last year. Notwithstanding the increase in the number of patients, expenses decreased, the total expenditure amountinng to £12,505, leaving a balance to the general fund of £1,253. A special appeal was made for contributions for the completion of the home, an appeal which has the approval and support of the Princess of Wales, the patroness of the charity. It is proposed to erect a new wing, by which further and much-needed accommodation will be provided for both inmates and staff, as well as a recreation hall, where entertainments can be held. £12,000 is the amount required for the building and the endowment of the new beds therein. The foundation stone is to be laid on Tuesday, 30th inst., by Earl Amherst. It was announced that the completion fund had now reached £3,700, including two gifts of £1,000 each.

The report having been adopted, sanction was given for the presentation of a petition praying for the granting of a Royal Charter to enable difficulties connected with the holding of gifts of land to be overcome, and conferring other advantages; and this concluded the proceedings of the meeting.

